# Prognosis and Progression of ESCC Patients with Perineural Invasion

**DOI:** 10.1038/srep43828

**Published:** 2017-03-03

**Authors:** Guanghui Xu, Fan Feng, Zhen Liu, Shushang Liu, Gaozan Zheng, Shuao Xiao, Lei Cai, Xuewen Yang, Guocai Li, Xiao Lian, Man Guo, Li Sun, Jianjun Yang, Daiming Fan, Qun Lu, Hongwei Zhang

**Affiliations:** 1 State Key Laboratory of Cancer Biology, Division of Digestive Surgery, Xijing Hospital of Digestive Disease, Fourth Military Medical University, Xi’an, 710032, China; 2 State Key Laboratory of Military Stomatology, Department of Operative Dentistry and Endodontics, School of Stomatology, Fourth Military Medical University, Xi’an, 710032, China

## Abstract

Perineural invasion (PNI) has been recognized as a poor prognostic factor in several malignancies, but the definition and pathogenesis of PNI in esophageal squamous cell carcinoma (ESCC) remains to be defined. PNI was evaluated by H&E staining and S100 immunohistochemistry. The predictive value of PNI in the prognosis of ESCC patients was analyzed. PNI was evaluated *in vitro* and *in vivo*. A total of 54 specimens (17.88%) were defined as PNI-a and 99 specimens (32.78%) as PNI-b. S100 staining was superior to H&E staining for PNI detection (50.66% vs 27.15%, *P* < 0.001, κ = 0.506). Tumor depth (*P* = 0.001), tumor stage (*P* = 0.010), and vascular invasion (*P* < 0.001) were significantly associated with PNI. PIN-a and PNI-b had significant lower disease free survival (DFS) and disease specific survival (DSS) than PNI-0 patients, and the prognosis of PNI-b patients was significantly worse than PNI-a patients for DFS (*P* = 0.009). PNI was an independent predictor for DFS and DSS in ESCC as evaluated by univariate and multivariate analyses. ESCC cells could metastasize along the nerve *in vitro* and *in vivo*, and PNI was a dynamic process. S100 staining significantly improved the accuracy of PNI detection. PNI was associated with local recurrence and poor prognosis of ESCC patients.

The highest incidence rates of esophageal cancer are found in Southern and Eastern Africa as well as Eastern Asia, of which 90% of cases are esophageal squamous cell carcinoma (ESCC). Despite remarkable advances that have been achieved in the prevention, diagnosis and treatment of ESCC, the morbidity and mortality of this disease have only minimally changed over the last decade. Metastasis and recurrence remain major causes of the poor prognosis of ESCC^1^. It is well known that tumor cells mainly metastasize through direct dissemination, vascular or lymphatic channels. However, the migration of tumor cells through perineural invasion (PNI) has been demonstrated as a new type of metastasis in recent years. PNI is regarded as a critical pathologic feature of metastasis through the perineurial system, which facilitates the spread and aggressive growth of tumor cells in distant locations^2^.

PNI is associated with poor prognosis of several malignancies including prostate cancer^3^, colorectal cancer^4^, head and neck cancer^5^ and gastric cancer^6^. However, the findings on the prognostic value of PNI in ESCC are limited and controversial^7^,^8^,^9^. In addition, the incidence of PNI in the same type of cancer varies considerably in that there are many factors that may influence the accuracy of PNI diagnosis, including the assessment method and lack of a precise definition. PNI has been characterized in several reports as the observation of tumor cells inside the perineurium layer^3^,^10^,^11^. However, Catherine Liebig^2^ characterized PNI as a tumor in close proximity to a nerve and involving at least 33% of its circumference or tumor cells within any of the 3 layers of the nerve sheath. In the majority of reports, PNI has been assessed by H&E staining. However, some investigators have used S100 immunohistochemistry to detect nerve fibers. Moreover, the progression of PNI in ESCC remains unknown.

Given this situation, the present study aimed to uncover the pathological features of PNI and to investigate the prognostic value of PNI in ESCC patients. We further investigated the progression of PNI in ESCC via *in vitro* and *in vivo* models.

## Results

### Histologic and ultrastructural findings of PNI

**Figure 1:**
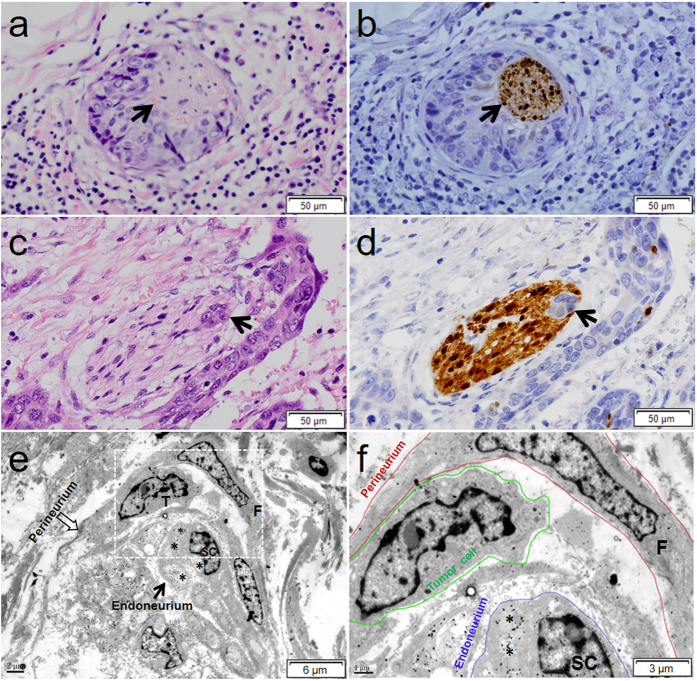
Representative histology and ultrastructure of PNI in ESCC. (**a**) The nerve fiber was partly embedded by tumor cells and stained by H&E. (**b**) The nerve fiber was identified in the serial section of A by positive S100 immunohistochemistry (arrow). The arrows indicate the nerve fiber at the same position. (**c**,**d**) Cancer cells (arrows) were identified within the perineurium of the nerve. (**e**) The axon (asterisk) is robustly surrounded by Schwann cells (SC), which constitutes the endoneurium of the nerve (arrow), ×4000. (**f**) View of the box in (**e**). The perineurium (red circle) is composed of fibroblasts (F), and tumor cells (green circle) have invaded the perineurium. A gap (arrow) exists between the esophageal cancer cells and the surrounding structures, ×10000.

To more precisely define PNI in ESCC, a grading system for PNI-a, PNI-b and PNI-0 was introduced in the current study. According to the definition of PNI, Fig. 1b was identified as PNI-a, and Fig. 1d was identified as PNI-b. To evaluate the influence of staining methods on PNI detection, we investigated PNI with H&E staining and S-100 immunohistochemical staining. In the majority of specimens, the edge of the nerve fascicle was unclear under H&E staining, while it was easily identified by S-100 immunohistochemical staining. PNI (PNI-a and PNI-b) was detected in 27.15% (82 of 302) and 50.66% (153 of 302) of cases with H&E staining and S-100 staining, respectively. We found that the PNI status determined in S100-stained sections was superior to the determination of PNI status in H&E-stained sections (McNemar test, *P* < 0.001, κ = 0.506) (Supplementary Table S1). As shown in Fig. 1a,c, the outline of the nerve fascicle was unclear under H&E staining, while it was easily identified by immunohistochemical staining with S100 (Fig. 1b,d).

To confirm the histological findings of PNI in ESCC, an ultrastructural examination of PNI was performed by immuno-electron microscopy. Tumor cells (T) were distributed in the perineurium (Fig. 1e). As shown in Fig. 1f, the perineurium is constituted of fibroblasts (F), and tumor cells (green circles) were located in the perineurium (red circles). Immune-gold particles, indicative of positive S100 immunostaining, were distributed in the Schwann cells (SC). The axons (asterisks) were surrounded by Schwann cells (SC), which constitute the endoneurium (blue circles).

### Correlation between PNI and clinicopathological characteristics

The clinicopathological characteristics of the ESCC patients are shown in Table 1. There were 233 males and 69 females. The median age was 58.4 years (range 34–79 years). Among them, 54 specimens (17.89%) were graded as PNI-a, and 99 specimens (32.78%) were graded as PNI-b. The remaining 149 specimens were graded as PNI-0. Tumor depth (*P* = 0.001), tumor stage (*P* = 0.010), and vascular invasion (*P* < 0.001) were significantly associated with PNI grade.

### Prognostic value of PNI

**Figure 2:**
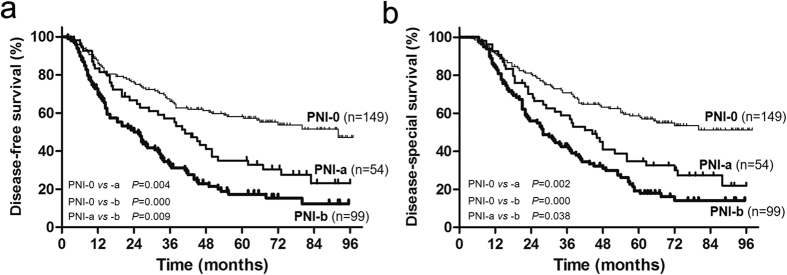
Prognosis of ESCC patients. Disease-free survival (**a**) and disease-specific survival (**b**) according to different types of PNI.

The five-year disease free survival (DFS) rates of PNI-0, PNI-a and PNI-b were 58.1%, 34.9%, and 17.3%, respectively (*P*_PNI-0 VS PNI-a_ = 0.004, *P*_PNI-0 VS PNI-b_ = 0.000, *P*_PNI-a VS PNI-b_ = 0.009, Fig. 2a). The five-year disease specific survival (DSS) rates of PNI-0, PNI-a and PNI-b were 58.7%, 38.9%, and 19.1%, respectively (*P*_PNI-0 VS PNI-a_ = 0.002, *P*_PNI-0 VS PNI-b_ = 0.000, *P*_PNI-a VS PNI-b_ = 0.038, Fig. 2b). Univariate analysis showed that differentiation status, tumor depth, lymph node metastasis, tumor stage, vascular invasion and PNI were predictors of DFS, and only differentiation status and PNI were independent prognostic predictors according to multivariate analysis (Table 2). Differentiation status, tumor depth, lymph node metastasis, lymph node metastasis, tumor stage, vascular invasion and PNI were predictors of DSS according to univariate analysis, and only differentiation status, vascular invasion and PNI were independent prognostic predictors (Table 3).

### Tumor cell migration along nerves *in vitro*

**Figure 3:**
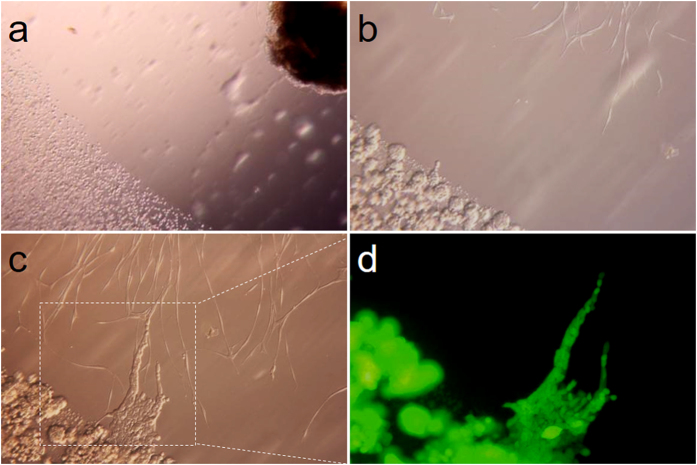
*In vitro* co-culture model of cancer cell colonies and nerve. (**a**–**c**) Dynamic process of the co-culture between cancer and DRGs in Matrigel (**a**: magnification, x40; **b** and **c**: magnification, x200). (**d**) GFP-labeled tumor cells travelled through neurites, which shows the same location as in *c* (magnification, x200).

To investigate the phenomenon of PNI in ESCC, a co-culture model of EC109 cell colonies transfected with green fluorescent protein (GFP) and dorsal root ganglia (DRGs) was used in present study. As shown in Fig. 3b, neurites of DRGs grew out in the direction of EC109-GFP cells on the 4^th^ day. Then, the migration of tumor cells along the neurites in the direction of DRGs was observed on the 7^th^ day (Fig. 3c). This experiment demonstrated crosstalk between cancer cells and nerve fibers. The neurites extended directionally and projected towards the tumor cell colonies. In addition, cancer cells dissociated from colonies and formed protrusions toward DRGs and then rapidly migrated along the neurites to DRGs once the cancer cells contacted neurites. Notably, tracing cells labeled with GFP demonstrated that the cells that migrated along the nerve were spindle-like in shape, while the cells that did not migrate maintained a squamous cell shape under fluorescence microscope (Fig. 3d). These findings suggested that tumor cells could metastasize along neurites.

### Patterns of perineural invasion in ESCC

**Figure 4:**
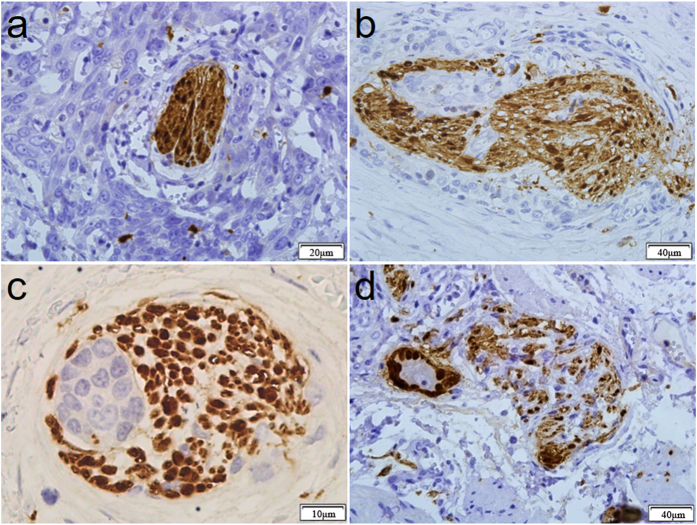
Variety patterns of the histologic appearance of PNI in ESCC. (**a**) The nerve fiber was tightly surrounded by tumor cells. The perineurium of the nerve exhibited high integrity. (**b**) Tumor cells invaded the nerve and damaged the perineurium of the nerve. (**c**) Tumor cells invaded the nerve. (**d**) The nerve fibers were irregularly damaged by tumor cells, which broke through the perineurium and grew outward.

In observing PNI, we noticed that the patterns of PNI were variable. As shown in Fig. 4a, the nerve was closely surrounded by tumor cells, and the perineurium was intact. As shown in Fig. 4b, the tumor cells damaged the perineurium and invaded into the nerve fascicle. Fig. 4c shows that tumor cells grew inside the nerve fascicle, and the perineurium was intact. Fig. 4d shows that the nerve fascicle was invaded and finally destroyed by tumor cells.

### Tumor cell migration along nerves *in vivo*

**Figure 5:**
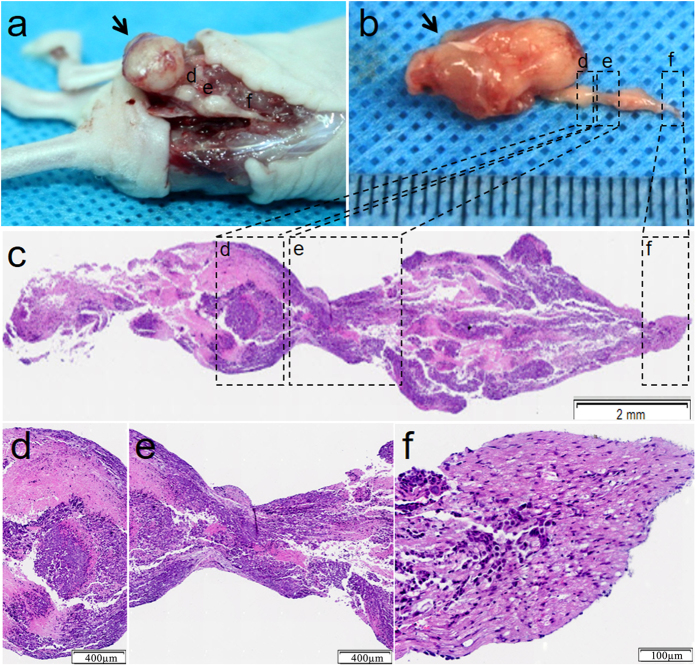
*In vivo* model of tumor metastasis along sciatic nerves. (**a**) A sciatic nerve injected with EC109. Arrows indicate the tumor implantation sites; arrowheads indicate the proximal nerve to the spine. (**b**) Isolated image of the sciatic nerve of Figure a. (**c**) Longitudinal pathological examination of the sciatic nerve by H&E staining. (**d–f**) Magnified area in the boxes of Figure c.

To further explore the patterns of ESCC metastasis, cancer cell metastasis in sciatic nerves was investigated using an *in vivo* animal model. As shown in Fig. 5a,b, a large carcinoma node was found at the primary injection point of the sciatic nerve. Moreover, small nodes were found at certain distances beyond the primary tumor injection point. To investigate the sciatic content in these small nodes, pathological examination of the nerves was performed. As shown in Fig. 5c, the nerve fascicle was filled with tumor cells with continuous migration along the nerve.

## Discussion

PNI is an under-recognized route of neoplastic invasion in a variety of malignancies. Investigation of the pathologic features and prognostic value of PNI has been reported for a variety of tumors, and the findings are controversial^12^,^13^,^14^. In the present study, PNI was confirmed in ESCC, and we found that PNI was a dynamic process. S100 staining could significantly improve the accuracy of PNI detection. PNI was associated with local recurrence and poor prognosis of ESCC patients.

It has been reported that the incidence of PNI in ESCC varied from 22.2% to 48.3%^7^,^15^. Non-standardized detecting methods may have contributed to the differences in the reported incidences of PNI. In the majority of reports, H&E staining was used to assess PNI^4^,^15^,^16^,^17^,^18^,^19^. However, determination of PNI is difficult and tedious by H&E if nerve fibers are severely damaged by invading tumor cells or if the nerve fibers are too thin. Thus, some investigators have used S100 immunohistochemistry to detect nerve fibers^20^,^21^. In the current study, both H&E staining and S100 staining were used to precisely identify PNI. Consistent with previous studies^20^,^22^, the results showed that S100 staining significantly improved the accuracy of PNI detection.

In addition, the differences in reported incidences of PNI may be partly due to the different definitions. Batsakis *et al*.^23^ proposed a broad definition of PNI in 1985, characterizing it as tumor cell invasion in, around and through nerves. Many investigators have proposed that at least 33% of the circumference of the nerve should be surrounded by tumor cells to consider it PNI^2^,^24^. In the present study, PNI was classified into 3 groups: PNI-a, PNI-b and PNI-0. PNI was significantly related to tumor depth, tumor stage and vascular invasion in the present study. We found that the prognosis of patients with PNI was significantly lower than that of patients without PNI. Moreover, both PNI-a and PNI-b were independent prognostic factors for DFS, which indicated that PNI was associated with an increased risk of local recurrence and reduced disease-free survival. We performed a detailed evaluation of the histopathologic quantitative characteristics of PNI to evaluate whether PNI-a and PNI-b can improve risk stratification in terms of DSS and DFS. We showed that the risk of worse DFS and DSS for PNI-b patients was significantly increased compared to patients with PNI-a. Herein, the quantitative characteristics of PNI should be considered while selecting parameters for risk stratification.

We presumed that PNI might be a dynamic process and include the following processes: 1. tumor cells promote axonogenesis and neurogenesis in tumor tissue, which was reported in our previous study^25^; 2. tumor cells grow surrounding the nerve fiber; 3. tumor cells invade the nerve sheath; 4. tumor cells migrate along the nerve; and 5. new metastatic foci are formed. Thus, we considered PNI-a and PNI-b as different stages of the same pathological process. PNI-a and PNI-b may coexist in one tumor. Therefore, PNI-b could be underestimated as PNI-a.

Our understanding of the pathogenesis of PNI has been limited due to the lack of effective models of the complex interaction between the nerve, tumor cells and extra-cellular microenvironment. *In vitro* models of DRGs and tumor cell co-cultures in Matrigel matrix cannot simulate the neural microenvironment. However, *in vivo* models are more promising and have been proven to be more effective. Therefore, an animal model of tumor cell invasion and migration along the sciatic nerve was investigated in the present study. We found that ESCC cells not only proliferate and invade the sciatic nerve at the site of injection but also metastasize along the nerve fascicle and form a neoplasm at a certain distance. This reflected the process of PNI to some extent. Recent studies have demonstrated that PNI may involve reciprocal signaling interactions between tumor cells and nerves^14^,^26^. On one hand, the increased neurite formation suggests that axonal migration may be a key element of PNI. Axonal growth is a complex process that requires neurotrophic growth factors and axonal guidance molecules. It is their potent effects on neuronal growth that have made neurotrophins prime candidates for studying the PNI invasion pathway. On the other hand, neurotransmitters and neuropeptides secreted by nerve terminations act as molecular determinants and promote tumor invasion and metastasis in PNI. There is a growing body of literature that suggests that the innervation of tumors, specifically from the autonomic nervous system, is critical to cancer progression^27^.

There are some limitations of the present study. First, the retrospective design in the single center might lead to some selection bias. Second, the sample size was not large. Third, the molecular mechanism of PNI was not investigated in the present study. These issues require further investigation.

In conclusion, PNI is a dynamic process. S100 staining can significantly improve the accuracy of PNI detection. PNI was associated with local recurrence and poor prognosis of ESCC patients.

## Materials and Methods

### Patients

This study was performed at the Xijing Hospital of Digestive Diseases affiliated with the Fourth Military Medical University. This study was performed as a retrospective cohort study at our institution in patients who underwent an esophagectomy for cancer between September 2008 and June 2011. A total of 302 ESCC patients were enrolled in the present study. The inclusion criteria were patients who 1. underwent minimally invasive esophagectomy; 2. had no neoadjuvant chemotherapy; and 3. had no distant metastasis. Patients were excluded if any one of the following exclusion criteria was present: 1. accompanied by a serious illness of the heart, brain or kidney; 2. previous esophageal surgery; 3. history of other malignancies; or 4. preoperative radiotherapy or chemotherapy. 1. accompanied by a serious illness of the heart, brain or kidney; 2. previous esophageal surgery; 3. history of other malignancies; or 4. preoperative radiotherapy or chemotherapy. The operation procedures were based on the Pittsburgh technique with slight modification^28^. All patients had undergone thoracoscopic esophageal mobilization and lymphadenectomy, laparoscopic gastric mobilization, lymphadenectomy and gastric conduit formation, and cervical anastomosis with cervical lymphadenectomy. Clinicopathological data including gender, age, tumor location, tumor size, pathological type, tumor depth, lymph node metastasis, tumor stage and lymphatic-vascular invasion were collected. The tumors were staged according to the seventh edition of the American Joint Committee on Cancer Tumor Node Metastasis (TNM) classification. All procedures followed were in accordance with the ethical standards of the responsible committee on human experimentation (The Ethics Committee of Xijing Hospital, China) and according to the Helsinki Declaration of 1964 and later versions. Informed consent was obtained from all patients upon inclusion in the study. The patients received postoperative chemotherapy and radiotherapy according to NCCN guidelines of esophageal cancer. The patients were followed up until November 2016 by enhanced CT every 3 months to evaluate tumor recurrence and metastasis.

### H&E and immunohistochemical staining

The tissues were fixed with 4% formaldehyde, embedded in paraffin and sectioned serially at 4 μm thickness. The sections were stained with either hematoxylin-eosin or anti-S100 antibody for immunohistochemistry as described previously^29^. Briefly, after antigen retrieval and blocking with normal goat serum, sections were allowed to react overnight at 4 °C with mouse monoclonal anti-S100 antibody (1:300, Abcam, USA). After incubating with the appropriate secondary antibody and DAB reaction, the slides were assessed via microscopy by 3 different pathologists.

### Definition of PNI

PNI-a: tumor cells were close to the nerve and involved in at least 33% of nerve’s circumference, or the tumor cells were infiltrated into the epineurium of the nerve sheath but not into the perineurium. PNI-b: tumor cells were infiltrated into the perineurium layer of the nerve sheath. PNI-0: no PNI-a or PNI-b was observed.

### Pre-embedding immunogold-silver cytochemistry

Immunogold-silver cytochemistry was performed in selected ESCC tissues using the method described previously^30^. Briefly, the tissues were immediately dissected and fixed in a mixture of 4% paraformaldehyde and 0.05% glutaraldehyde in 0.1 M PBS for 24 h at 4 °C. Serial sections of 50 μm thickness were prepared using a vibratome. The sections were placed in 25% sucrose and 10% glycerol for 1 h. After freeze-thaw treatment, the sections were immersed in PBS containing 5% normal goat serum and then incubated overnight with primary mouse anti-S100 antibody (1:300, Abcam, USA). After washing in PBS, the secondary anti-mouse IgG conjugated to 1.4 nm gold particles at a 1:100 dilution was applied (Nanoprobe, Stony Brook, NY) and followed by post-fixation in 2% glutaraldehyde for 45 min. Silver enhancement was performed in the dark using a HQ Silver Kit. Tissues were then incubated in ABC solution and visualized by the glucose oxidase-3,3′-diaminobenzidine method. Immunolabeled sections were fixed with 0.5% osmium tetroxide for 1 h, dehydrated in a graded ethanol series followed by propylene oxide, and finally flat-embedded in Epon812. Sections labeling S100 immunoreactivity were selected, trimmed under a stereomicroscope, and mounted onto blank resins tubs. Ultrathin sections were cut using an Ultramicrotome (EMUC6, Leica) and mounted on mesh grids. They were then counter-stained with uranyl acetate and lead citrate and observed under a JEM-1230 electron microscope (JEOL LTD, Tokyo, Japan).

### Tumor cell metastasis along the nerve *in vitro*

Sprague-Dawley rats 4 weeks of age (provided by Animal Center of the Fourth Military Medical University) were sterilized with 75% alcohol and euthanized with CO_2_. Dorsal root ganglia (DRGs) were dissected from lumbar areas under sterile conditions. On primary isolation, DRGs were kept on ice in artificial cerebrospinal fluid^31^. The spinal dura mater and pia mater were removed from the DRGs under a stereoscopic dissecting microscope (Olympus, Japan). The prepared DRGs were seeded on 13-mm plastic cover slips (Millipore) and then covered with 20 μl extracellular Matrigel (BD, USA). Subsequently, 1 × 10^5^ EC109-GFP cells suspended in 20 μl Matrigel were placed at a 3-mm distance adjacent to the DRGs. In addition, 30 μl additional Matrigel was dropped at the distance of the DRGs and tumor cells in order to build a steady and physiological environment in *vitro*. The coverslips were laid in a 6-well plate and placed in an incubator for 20 min. The coverslips were cautiously submersed in DMEM-F12 (HyClone, USA) supplemented with 10% fetal bovine serum (GIBCO). This co-culture model was continuously incubated in a humidified atmosphere of 5% CO_2_ at 37 °C and observed under a light or fluorescence microscope.

### Tumor cell metastasis along the nerve *in vivo*

An *in vivo* model of cancer cell metastasis was prepared as published previously^32^. Four-week-old athymic nude mice were deeply anesthetized with 10% chloral hydrate (0.04 ml/10 g) by intraperitoneal injection. The sciatic nerves were identified at the femoral coccygeus and biceps femoris muscles. Then, 3 × 10^5^ EC109 cells were microscopically injected around the perineurium of the sciatic nerves of the anesthetized nude mice distal to the bifurcation of the tibial and common peroneal nerves. The sciatic nerve was stained with H&E at the time of death.

### Statistical analysis

The data were processed using SPSS 22.0 for Windows (SPSS Inc., Chicago, IL, USA). Discrete variables were analyzed using Chi-square tests or Fisher’s exact tests. Significant risk factors identified by univariate analysis were further assessed by multivariate analysis using the Cox’s proportional hazards regression model. To compare the results determined based on different staining methods, paired McNemar tests were performed, and κ values were calculated to reflect the agreement between the two staining methods. DFS and DSS were analyzed by the Kaplan-Meier method. *P* values were considered statistically significant at the 5% level.

## Additional Information

**How to cite this article:** Xu, G. *et al*. Prognosis and Progression of ESCC Patients with Perineural Invasion. *Sci. Rep.* **7**, 43828; doi: 10.1038/srep43828 (2017).

**Publisher's note:** Springer Nature remains neutral with regard to jurisdictional claims in published maps and institutional affiliations.

## Supplementary Material

Supplementary Table S1

## Figures and Tables

**Table 1:** Relationship between PNI and clinicopathological characteristics of ESCC patients.

Characteristics	n	PNI			*P* value
		PNI-0 (149)	PNI-a (54)	PNI-b (99)	
Age					0.722
<59	162	79 (48.77%)	27 (16.67%)	56 (34.57%)	
≥ 59	140	70 (50.00%)	27 (19.29%)	43 (30.71%)	
Gender					0.386
Male	233	110 (47.21%)	44 (18.88%)	79 (33.91%)	
Female	69	39 (56.52%)	10 (14.49%)	20 (28.99%)	
Pain					0.778
Negative	224	112 (50.00%)	38 (15.57%)	74 (33.04%)	
Positive	78	37 (47.44%)	16 (20.51%)	25 (32.05%)	
Location					0.840
Upper	50	25 (50.00%)	10 (20.00%)	15 (30.00%)	
Middle	146	76 (52.05%)	24 (16.44%)	46 (31.51%)	
Lower	106	48 (45.28%)	20 (18.87%)	38 (35.85%)	
Tumor size					0.646
≤3 cm	113	58 (51.33%)	22 (19.47%)	33 (29.20%)	
3–5 cm	108	56 (51.85%)	17 (15.74%)	35 (32.41%)	
≥ 5 cm	81	35 (43.21%)	15 (18.52%)	31 (38.27%)	
Differentiation					0.232
Well	153	75 (49.02%)	27 (17.65%)	51 (33.33%)	
Moderate	114	56 (49.12%)	25 (21.93%)	33 (28.95%)	
Poor	35	18 (51.43%)	2 (5.71%)	15 (42.86%)	
Tumor depth					0.001
T1	49	36 (73.47%)	7 (14.29%)	6 (12.24%)	
T2	97	49 (50.52%)	18 (18.56%)	30 (30.93%)	
T3 + T4	156	64 (41.03%)	29 (18.59%)	63 (40.38%)	
Lymph node metastasis					0.089
N0	165	92 (55.76%)	29 (17.58%)	44 (26.67%)	
N1	101	43 (42.57%)	20 (19.80%)	38 (37.62%)	
N2	20	10 (50.00%)	2 (10.00%)	8 (40.00%)	
N3	16	4 (25.00%)	3 (18.75%)	9 (56.25%)	
Tumor stage					0.010
I	86	52 (60.47%)	14 (16.28%)	20 (23.26%)	
II	120	61 (50.83%)	24 (20.00%)	35 (29.17%)	
III	96	36 (37.50%)	16 (16.67%)	44 (45.83%)	
Vascular invasion					0.000
Negative	176	121 (68.75%)	23 (13.07%)	32 (18.18%)	
Positive	126	28 (22.22%)	31 (24.60%)	67 (53.17%)	

**Table 2:** Univariate and multivariate analysis of the prognostic factors for disease-free survival of ESCC patients.

Characteristics	Univariate analysis				Multivariate analysis	
	β	HR (95% CI)	*P*value	β	HR (95% CI)	*P* value
Age	−0.028	0.972 (0.727–1.300)	0.849			
<59/≥59						
Gender	−0.216	0.805 (0.561–1.156)	0.240			
Male/Female						
Pain	0.337	1.401 (0.983–1.996)	0.062			
Negative/Positive						
Location	0.042	1.042 (0.841–1.292)	0.705			
Upper/Middle/Lower						
Tumor size	0.137	1.146 (0.955–1.377)	0.143			
≤3/3–5/≥5 cm						
Differentiation	0.289	1.335 (1.071–1.663)	0.010	0.311	1.365 (1.089–1.711)	0.007
Well/Moderate/Poor						
Tumor depth	0.414	1.512 (1.224–1.869)	0.000	0.198	1.219 (0.930–1.597)	0.151
T1/T2/T3 + T4						
Lymph node metastasis	0.441	1.554 (1.161–2.081)	0.003	−0.037	0.963 (0.619–1.499)	0.868
N0/N1/N2/N3						
Tumor stage	0.399	1.491 (1.230–1.807)	0.000	0.189	1.208 (0.865–1.685)	0.267
I/II/III						
Vascular invasion	0.729	2.073 (1.548–2.777)	0.000	0.320	1.376 (0.984–1.926)	0.062
Negative/Positive						
PNI	0.522	1.685 (1.429–1.987)	0.000	0.410	1.506 (1.248–1.818)	0.000
PNI-0 *vs* PNI-a	0.597	1.816 (1.218–2.708)	0.003	0.415	1.515 (0.992–2.312)	0.034
PNI-0 *vs* PNI-b	1.046	2.846 (2.041–3.969)	0.000	0.820	2.270 (1.555–3.314)	0.000
PNI-a *vs* PNI-b	−0.449	0.638 (0.432–0.942)	0.024	−0.405	0.667 (0.450–0.989)	0.044

**Table 3:** Univariate and multivariate analysis of the prognostic factors for disease-specific survival of ESCC patients.

Characteristics	Univariate analysis				Multivariate analysis	
	β	HR (95% CI)	*P* value	β	HR (95% CI)	*P*value
Age	−0.013	0.987 (0.738–1.320)	0.931			
<59/≥59						
Gender	−0.226	0.798 (0.556–1.144)	0.798			
Male/Female						
Pain	0.337	1.400 (0.982–1.996)	0.063			
Negative/Positive						
Location	0.062	1.064 (0.858–1.319)	0.572			
Upper/Middle/Lower						
Tumor size	0.147	1.158 (0.964–1.391)	0.117			
≤3/3–5/≥5 cm						
Differentiation	0.316	1.371 (1.099–1.710)	0.005	0.336	1.399 (1.116–1.755)	0.004
Well/Moderate/Poor						
Tumor depth	0.429	1.536 (1.242–1.898)	0.000	0.209	1.232 (0.940–1.616)	0.131
T1/T2/T3 + T4						
Lymph node metastasis	0.481	1.618 (1.208–2.166)	0.001	0.015	1.015 (0.654–1.578)	0.946
N0/N1/N2/N3						
Tumor stage	0.423	1.527 (1.258–1.853)	0.000	0.184	1.202 (0.862–1.677)	0.278
I/II/III						
Vascular invasion	0.724	2.063 (1.540–2.764)	0.000	0.342	1.407 (1.010–1.961)	0.044
Negative/Positive						
PNI	0.496	1.642 (1.394–1.934)	0.000	0.376	1.456 (1.209–1.755)	0.000
PNI-0 *vs* PNI-a	0.622	1.863 (1.249–2.779)	0.002	0.433	1.542 (1.010–2.352)	0.045
PNI-0 *vs* PNI-b	0.996	2.707 (1.944–3.771)	0.000	0.757	2.132 (1.464–3.104)	0.000
PNI-a *vs* PNI-b	−0.374	0.688 (0.466–1.015)	0.060	−0.324	0.723 (0.488–1.071)	0.105
