# Supplementary material for: Prognosis and Progression of ESCC Patients with Perineural Invasion
Source: Sci Rep. 2017 Mar 3;7:43828. doi: 10.1038/srep43828 (PMC5335559; doi:10.1038/srep43828)
Supplement: Supplementary Table S1 [file srep43828-s1.doc]

Prognosis and Progression of ESCC Patients with Perineural Invasion

Running head: Perineural Invasion in Esophageal Squamous Cell Carcinoma

Guanghui Xu^1,*^, Fan Feng^1,*^, Zhen Liu^1,*^, Shushang Liu^1^, Gaozan Zheng^1^, Shuao Xiao^1^, Lei Cai^1^, Xuewen Yang^1^, Guocai Li^1^, Xiao Lian^1^, Man Guo^1^, Li Sun^1^, Jianjun Yang^1^, Daiming Fan^1^, Qun Lu^2^ **&** Hongwei Zhang^1^

^1^State Key Laboratory of Cancer Biology, Division of Digestive Surgery, Xijing Hospital of Digestive Disease, Fourth Military Medical University, Xi’an, 710032, China

^2^ State Key Laboratory of Military Stomatology, Department of Operative Dentistry and Endodontics, School of Stomatology, Fourth Military Medical University, Xi’an, 710032, China

*These authors contributed equally to this work.

Correspondence and requests for materials should be addressed to H.Z. (email: zhanghwfmmu@126.com) and Q.L. (email: [luqun22@hotmail.com](mailto:luqun22@hotmail.com)).

Table S1 The comparison of S100 staining and H&E staining on detection of PNI.

|  | | S100 staining | | *P* value | Kapaa |
| --- | --- | --- | --- | --- | --- |
|  |  | Positive | Negative |  |  |
| H&E staining | Positive | 80 | 2 | <0.001 | 0.506 |
|  | Negative | 73 | 147 |  |  |
